# Dentist´s knowledge, attitudes and determining factors of the conservative approach in teeth with reversible pulpitis and deep caries lesions

**DOI:** 10.4317/jced.55395

**Published:** 2018-12-01

**Authors:** Isabel Crespo-Gallardo, Jenifer Martín-González, María C. Jiménez-Sánchez, Daniel Cabanillas-Balsera, Benito Sánchez-Domínguez, Juan J. Segura-Egea

**Affiliations:** 1 Endodontics Section, Department of Stomatology, (Conservative Dentistry Section), University of Sevilla, Spain

## Abstract

**Background:**

The aim of this study was to investigate dentists` knowledge, attitudes and factors regarding the conservative approach in the management of deep caries lesions (DCLs) in teeth with reversible pulpitis.

**Material and Methods:**

187 dentists were contacted directly or by mail, and 125 (67%) were finally included in the study. Dentists were requested to answer an open/discursive questionnaire about the routine approach to the diagnosis and treatment of DCLs, including knowledge-related attitudinal items.

**Results:**

Total caries excavation was the preferred treatment option for more than 80% of dentists in case of DCL with reversible pulpitis. Only a small percentage (8%) chose partial caries removal, leaving some carious dentin close to the pulp to avoid pulp exposure. More than a half (51%) of respondents considered that cariogenic microorganisms must be removed or caries would progress. Dentists teaching at the University strongly disagreed with this statement (OR = 4.6; 95% C.I. = 1.3 – 15.8; *p* = 0.017). Good clinical result was the most chosen reason (83%) to choose a specific treatment. Patient’s oral health (84%) and patient’s age (70%) were the two patient-related factors most taken into account for the choice of treatment.

**Conclusions:**

Total caries excavation is still the most frequently chosen treatment in teeth with DCL and reversible pulpitis. The joint assessment of the answers given by respondents allows to conclude that the new knowledge and concepts about caries lesions and the more conservative approach to DCLs have not still been incorporated by dentists into their usual clinical practice.

** Key words:**Caries, deep caries lesions, dental pulp capping, dental pulp health, dentists, endodontic therapy, reversible pulpitis, treatment decisions.

## Introduction

The conception of caries as an infectious disease meant that, until the end of the last century, the surgical treatment of the caries lesion consisted in the elimination of all the infected biomass, with replacement of the lost hard tissue. The new concept of caries as an ecological imbalance in the oral biofilm (1), and not as an infection, has led to a change in the approach to the treatment of caries lesions. Currently, the preservation of dental hard tissues, by mean of minimally invasive restorative treatments, takes precedence over their elimination (2). In the treatment of teeth with asymptomatic vital pulps and carious lesions involving, radiographically, the inner pulpal third of dentin (deep carious lesion, DCLs), the main objective should be to avoid pulp exposure, allowing tooth retention for long-term, and avoiding potentially painful, costly, and invasive endodontic treatments (3).

An important part of the clinical practice of dentists is to treat deep carious lesions (DCLs). Several studies have demonstrated that the diagnostic criteria and the therapeutic protocols applied by each dentist in the management of DCLs lesions are variable (4). The depth that is reached in the removal of carious dentin is especially variable, ranging from the non-selective removal of decayed tissue to hard dentin, with complete excavation of the decayed tissue, leaving only hard dentin, with high risk of pulp exposure, to selective caries removal, leaving soft dentin in the area of the cavity near the pulp (5,6). The International Caries Consensus Collaboration Group (ICCC), linked to the European Organization for Caries Research, the International Association for Dental Research-Cariology Group and the American Dental Education Association-Cariology Section, has established well-defined criteria for the treatment of DCLs (7,8). These criteria considered complete excavation or removal of carious dentin over-treatment DCLs (3,4,7-9). However, several surveys carried out in different countries indicate that some dentists continue this practice (5,6,10,11).

In Spain, as long as we know, no study has been published providing current data on the management of DCLs by dentists. Therefore, it is not known if dentists have incorporated minimally invasive approaches to the removal of decayed tissue in their usual clinical practice. The aim of this study was to conduct a survey investigating dentists` knowledge, attitudes and factors regarding the conservative approach to deep caries lesions in teeth with reversible pulpitis.

## Material and Methods

Ethical approval of this study was considered unnecessary by the Ethical Committee of the University of Sevilla.

-Selection and recruitment process

The survey was carried out in Sevilla (Andalucía, Spain) during 2017. A total of 187 dentists, randomly selected amongst those working or attending postgraduate courses in the Dental School of the University of Sevilla, with both private and public clinical practice, were contacted directly or by mail. One hundred and thirty four (71.6%) fulfilled the survey, being excluded 6 dentists because they answered the questionnaire incompletely and 3 dentists because they were no longer practicing clinical activities. Therefore, 125 (66.8%) dentists were included in the study. The purpose of the study was explained to all and indicated confidential and anonymous processing of the data.

-Questionnaire

**Figure 1:**
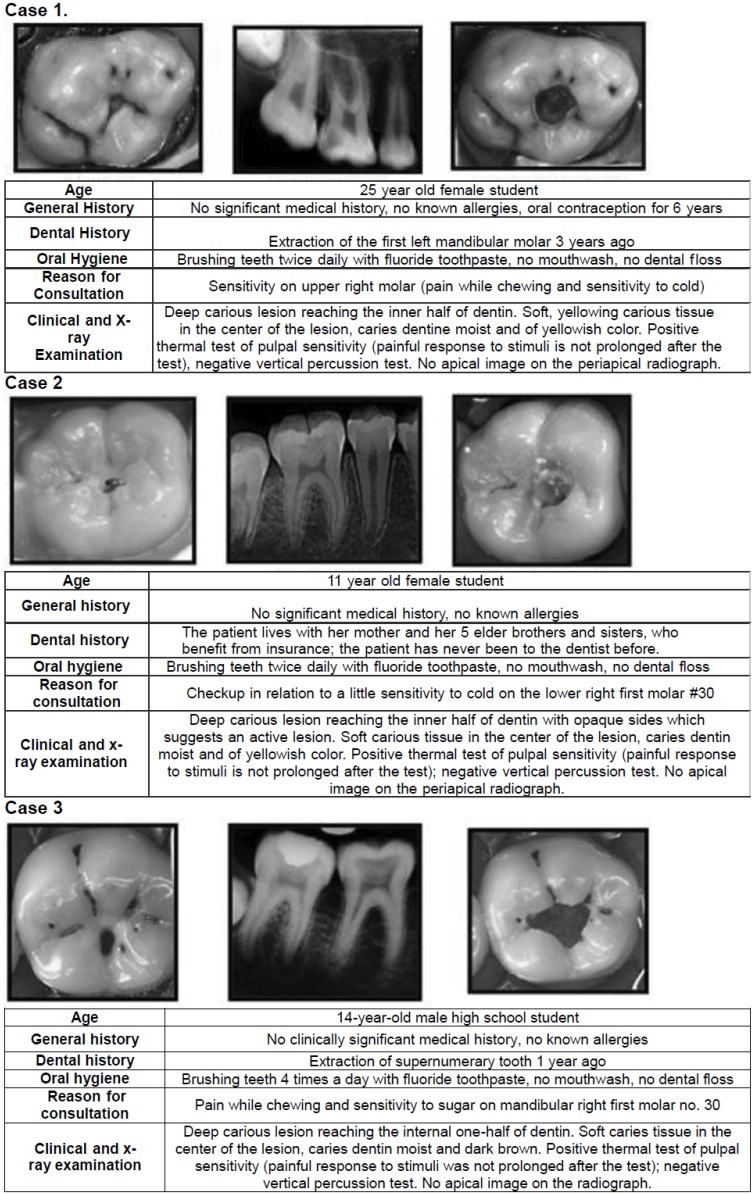
Overview of the information included in the 3 cases provided (Koopaeei *et al.* 2017) (5).

Respondent dentists were requested to answer an open/discursive questionnaire based in previous surveys conducted in Brazil (12), USA (5) and Europe (6,10,11) ( Table 1,  Table 1 continue,  Table 1 continue-1). Translation of the English master versions was performed by native speakers. After several questions concerning the respondents’ demographic, educational, and professional backgrounds, presented the respondents the same 3 clinical scenarios originally developed by Weber *et al.* (12) and also used by Koopaeei *et al.* (5) (Fig. 1). The simulated clinical cases were composed of young patients (ages 25, 11, and 14 for cases A,B, and C, respectively) with no medical history of allergies or use of medications, reporting the occurrence of pain provoked by chewing or by cold in posterior teeth as their main complaint (12). Each case included a clinical occlusal view, a periapical radiograph, and a clinical occlusal view after opening the lesion, plus information about the patient’s age, general and dental history, oral hygiene practices, and the reasons for the consultation and clinical radiographic examination. For each case, the respondents had to choose the most likely diagnosis, and then which treatment would be indicated. Respondents were also asked concerning routine approaches to treating DCLs (5), developed originally by Schwendicke *et al.* (10), and questions concerning the respondents’ reasons for their treatment preferences and knowledge-related attitudinal items (5), developed originally by Schwendicke *et al.* (10) and Stangvaltaite *et al.* (11).

**Table 1:**
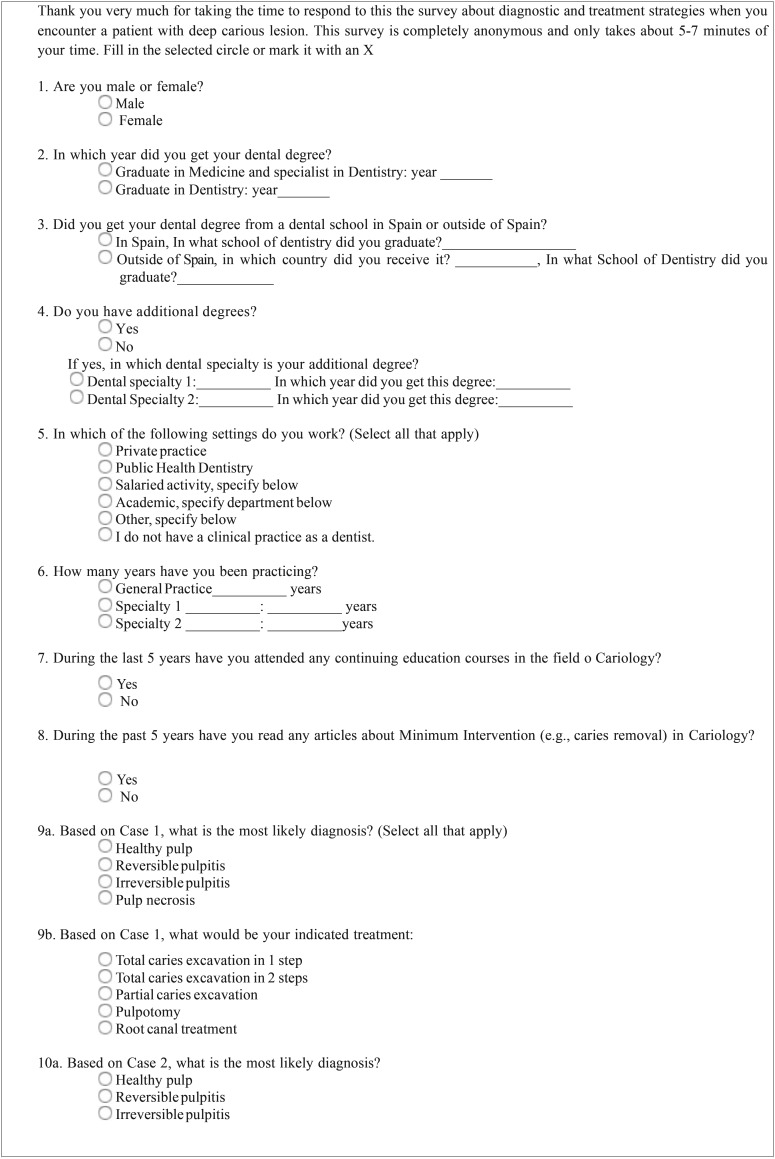
Questionnaire.

**Table 1 continue:**
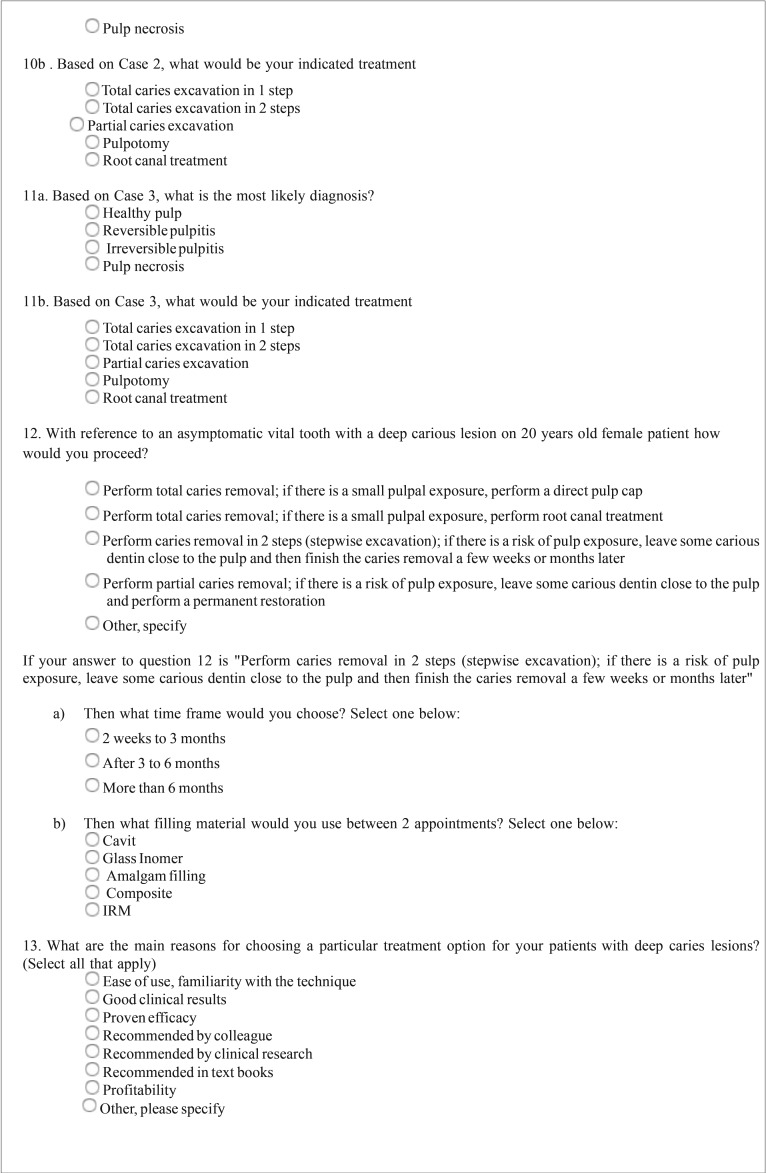
Questionnaire.

**Table 1 continue-1:**
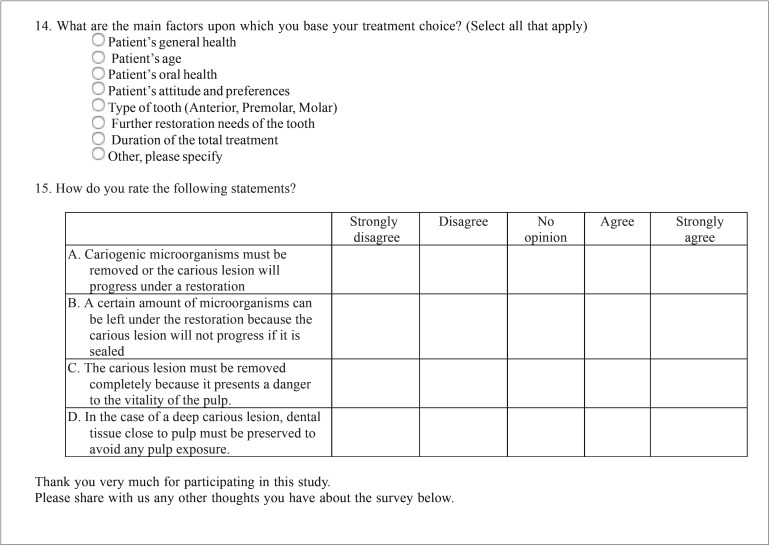
Questionnaire.

-Statistical analysis

A database was created for further analysis using Excel (Microsoft Corp., Redmond, WA, USA). Data description was carried out by frequency tables to provide an overview of the responses. When obtaining the numerical representation by percentages, the total number of answers for each query was taken into account. Logistic regression analysis was carried out transforming qualitative explanatory variables into binary variables. Explanatory variables were entered and then removed stepwise if *p* > 0.10 (hierarchical method). Odds ratios (OR) and confidence intervals (CI) were calculated as effect estimates. Significant differences were considered when *p* < 0.05.

## Results

-Participants’ characteristics

The survey was answered by 125 dentists, 34 men (27.2%) and 91 women (72.8%) ( Table 2). Of these, 73 (58.4%) had undergone specialized dental training. The average time of dental clinical practice was 7.1 years. The majority of the respondents worked in private practice (74.4%), and 45.6% had attended at least one continuing education course in cariology in the last 5 years. Ninety-seven (77.6%) had read an article about minimal intervention in the treatment of carious lesions.

**Table 2:**
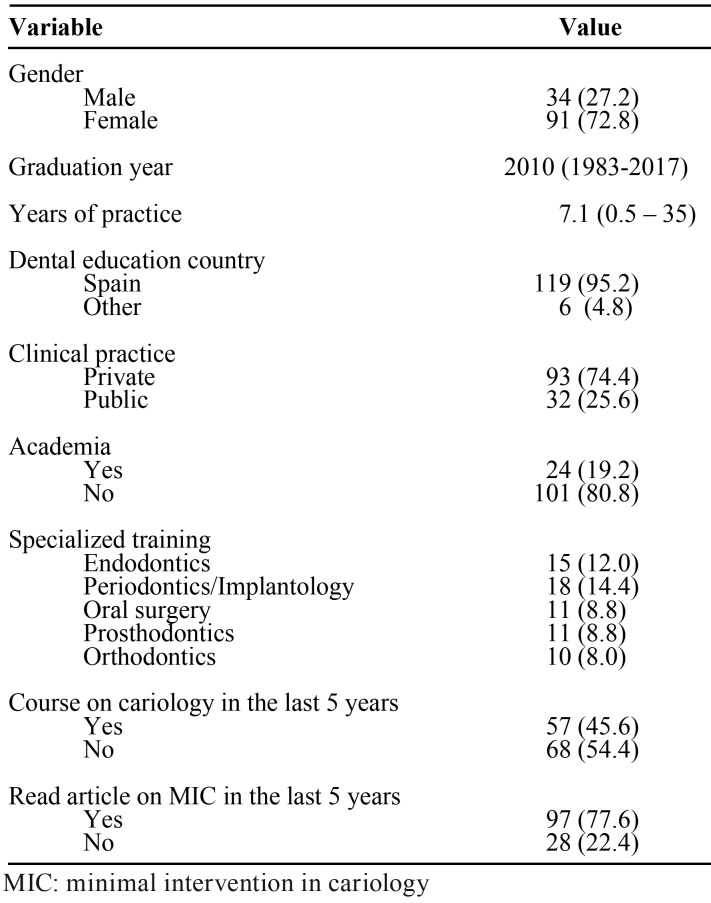
Demographic, academic and professional variables.

-Pulpal diagnoses and treatment options

Case 1

**Figure 2:**
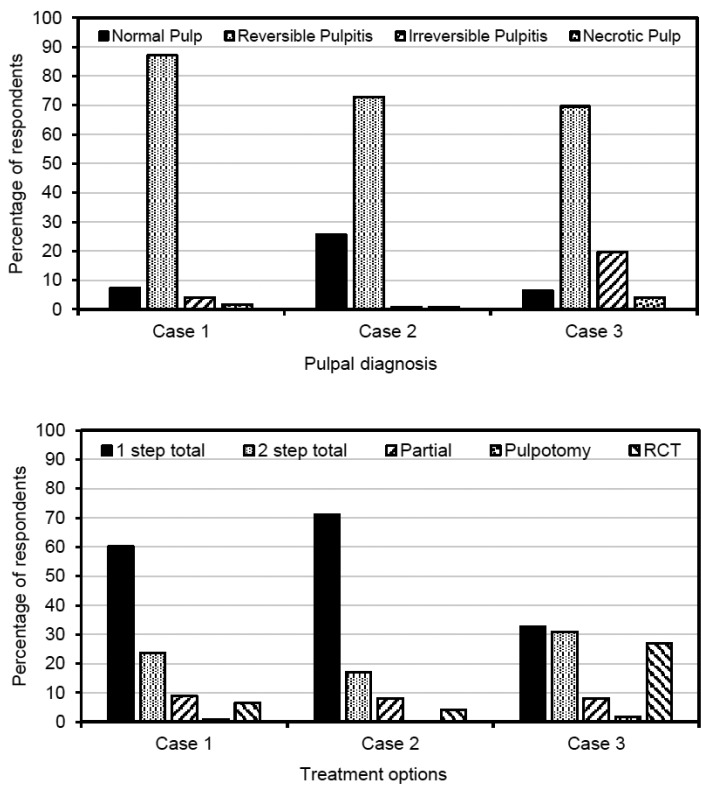
Pulpal diagnoses and treatment options selected by respondents for the 3 clinical case scenarios provided in the survey.

The answers to the 3 clinical cases are showed in figure 1. Regarding the pulpal diagnosis (Fig. 2, top), in case 1 reversible pulpitis was the most frequent diagnosis (87.1%). Total caries excavation was selected as the best treatment option by 83.8% of dentists, being one-step total caries excavation the modality most frequently chosen (60.2%) (Fig. 2, bottom).

Case 2

In case 2, 72.8% of respondents agreed on a diagnosis of reversible pulpitis, and 25.6% considered that the pulp was healthy. Concerning the treatment, 87.9% selected total caries excavation as the best option, choosing one-step and two-step 71.0% and 16.9%, respectively.

Case 3

In case 3, 69.7% of dentists chose reversible pulpitis as the diagnosis, and 19.7% agreed on irreversible pulpitis as a possible diagnosis. Most of respondents (63.4%) chose any of the types of total caries excavation, and more than a quarter of dentists (26.8%) indicated that it would perform in this case root canal treatment. Very few dentists (less than 2%) selected pulpotomy as a treatment option.

Approximately 8% of respondents in each of the 3 cases chose partial caries excavation as treatment option, being this option significantly more chosen by dentists who received courses in cariology in the last 5 years (OR = 3.1; 95% C.I. = 1.0 – 9.3; *p* = 0.047).

-Asymptomatic vital tooth with a DCL

**Figure 3:**
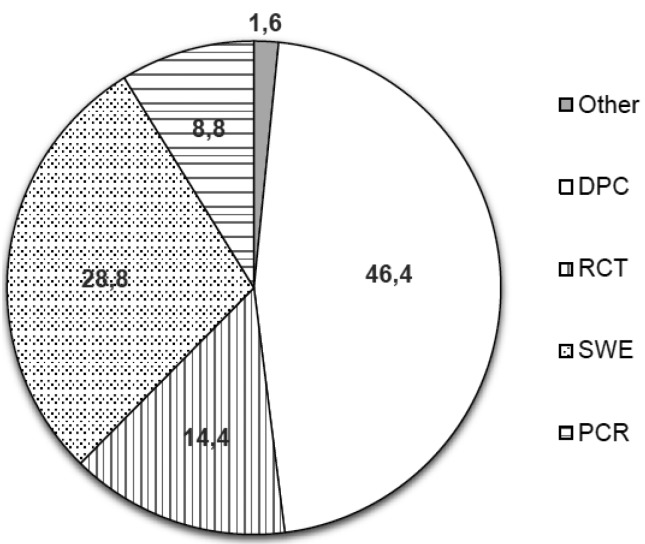
Treatment options selected by respondents for the case of an asymptomatic vital tooth with a DCL on 20 years old female patient. DPC: total caries removal and, if pulpal exposure occur, direct pulp capping; RCT: total caries removal and, if pulpal exposure occur, root canal treatment. SWE: stepwise excavation; PCR: partial caries removal.

Regarding the clinical situation raised in item 12 of questionnaire (an asymptomatic vital tooth with a DCL on 20 years old female patient) (Fig. 3), 60.8% of dentists chose to perform total caries removal and, if pulpal exposure occur, 46.4% and 14.4% would perform direct pulp capping or root canal treatment, respectively. Less than a third (28.8%) chose to perform caries removal in 2 steps (stepwise excavation) and, if there is a risk of pulp exposure, leave some carious dentin close to the pulp, and then finish the caries removal a few weeks or months later. Only 8.8% of respondents chose to perform partial caries removal and, if there is a risk of pulp exposure, leave some carious dentin close to the pulp and perform a permanent restoration. This last option was again significantly more chosen by dentists who received courses in cariology in the last 5 years (OR = 5.1; 95% C.I. = 1.0 – 25.8; *p* = 0.046).

-Knowledge and attitude on the management of deep carious lesions

Four assertions were included in the questionnaire regarding knowledge and aptitude on the management of deep carious lesions for which the respondents had to indicate agreement or disagreement on a 5-point scale ( Table 3). When asked if cariogenic microorganisms must be removed or caries would progress, 50.4% strongly agreed, and only 14.6% and 8.9% disagreed or strongly disagreed, respectively. The dentists who taught at the University strongly disagreed with this statement (OR = 4.6; 95% C.I. = 1.3 – 15.8; *p* = 0.017).

**Table 3:**
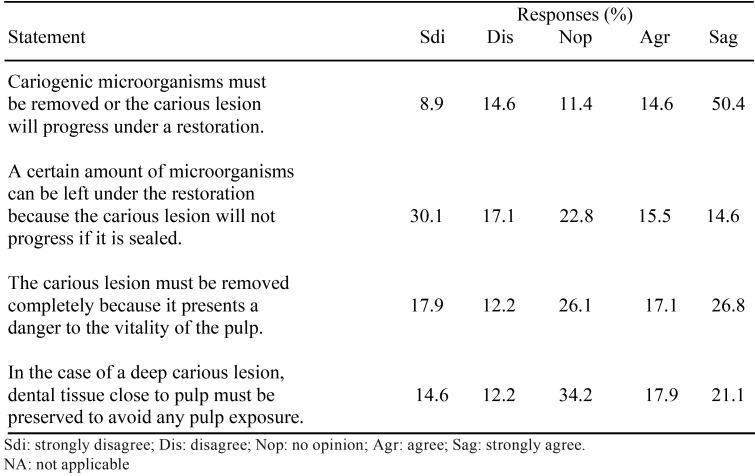
Knowledge-related attitudinal responses to management of deep carious lesions.

To the question if a certain amount of microorganisms could be left during caries removal, the answers were much divided, and only 15.5% and 14.6% agreed or strongly agreed, respectively. Dentists teaching at the University significantly agreed with this statement (OR = 4.5; 95% C.I. = 1.2 – 16.8; *p* = 0.0247).

When asked if a carious lesion must be removed to prevent its presence from damaging the vitality of the pulp, again the answers were very distributed, being the most frequent strongly agree (26.8%).

The fourth question asked if, treating a deep carious lesion, dental tissue close to the pulp must be preserved to avoid any pulp exposure, and again the opinions of the respondents were very variables, being “no opinion” the most frequent (34.2%).

-Factors influencing the treatment choice 

Finally, dentists were asked about their reasons to choose a specific treatment and about the factors influencing the treatment choice in managing deep carious lesions ( Table 4). The good clinical result was the most chosen reason (82.3%), and patient’s oral health (84.0%) and patient’s age (69.6%) were the two patient-related factors most taken into account for the choice of treatment.

**Table 4:**
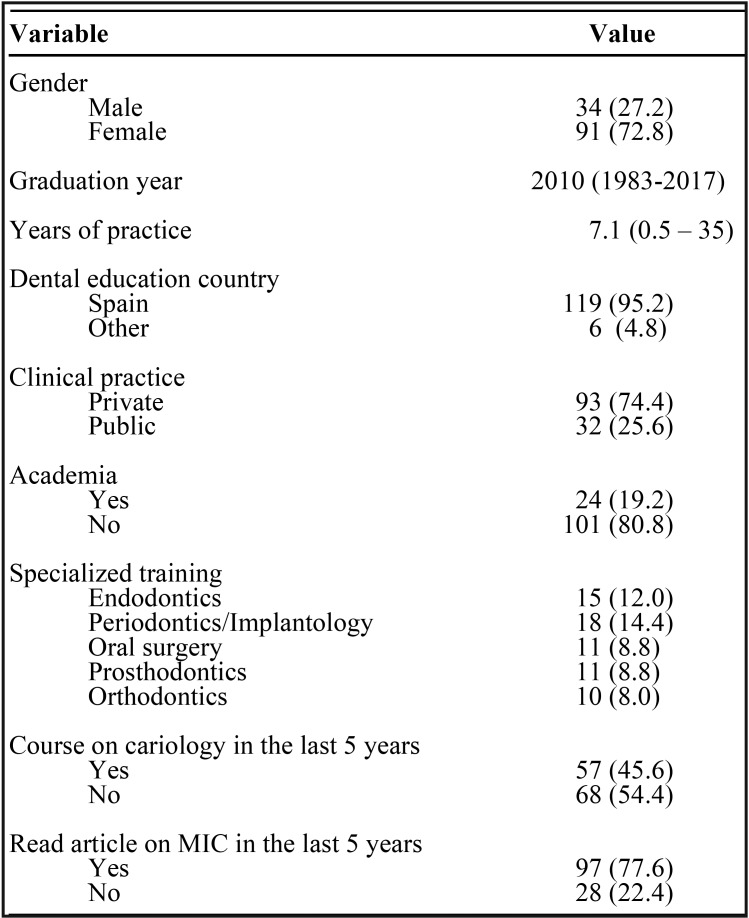
Percentage of yes responses for reasons for preferred treatment and main factor for treatment choice in managing deep carious lesions.

## Discussion

This study aimed to investigate dentists` knowledge, attitudes and decisions strategies in the management of DCLs. If the answers given by dentists are assessed globally, it can be concluded that dentists have not completely incorporated into their usual clinical practice the new therapeutic approach to caries lesions, which is more conservative and based on minimal intervention. Total caries excavation is the preferred treatment option in case of DCL with reversible pulpitis. Moreover, the results suggest that there is an excess indication of endodontic treatment.

Surveys are a valid method widely used to assess the knowledge, attitudes and decision strategies of dentists (5,10,13). Several surveys conducted in other countries have investigated dentist´s knowledge, attitudes and decisions strategies regarding DCLs, most of them including the same three clinical cases used in the present study and/or similar questions (5,6,10-12). The study sample was selected among general dental practitioner in Andalusia (southern Spain). Both the sample size and the response rate were comparable to those found in other surveys conducted previously (5,10,11). Seventy three per cent of the respondents were women, in agreement with the highest proportion of women who currently graduate in Spanish dental schools. In other surveys conducted in Spain and other countries, a similar feminization of samples has been observed (12,14,15).

The dentists had to answer several questions related to three clinical cases. The first and second clinical scenarios referred to asymptomatic patients with moderately DCLs not reaching the inner third of the dentin, being mostly diagnosed by respondents as reversible pulpitis. Concerning the treatment, total caries excavation (1 step or 2 steps) was the preferred options for more than 80% of dentists. Only a small percentage chose partial caries removal (8%) or RCT (5%). The same two clinical scenarios were used in previous surveys conducted in USA (5) and Brazil (12), being the percentages of answers, in both cases, similar to those of the present study, and preferring most of the respondents remove all the carious tissue. The third case referred to an asymptomatic patient with a DCL and no lingering pain to cold test, showing the radiograph a great radiolucent area into the tooth crown. Seventy percent of dentists diagnosed reversible pulpitis, but 20% chose irreversible pulpitis, being total caries excavation (63%), RCT (27%) and partial caries excavation (8%) the treatments options. In the same case, 35% of American dentists (5) and 7% of Brazilians (12) chose endodontic therapy. It was observed that dentists who received courses in cariology in the last 5 years were three times more likely (OR = 3.1; *p* = 0.047) to indicate a conservative treatment such as partial caries excavation, highlighting the importance of dental continuing education. Only 8.8% of respondents chose to perform partial caries removal and, if there is a risk of pulp exposure, leave some carious dentin close to the pulp and perform a permanent restoration. This last option was again significantly more chosen by dentists who received courses in cariology in the last 5 years (OR = 5.1; 95% C.I. = 1.0 – 25.8; *p* = 0.046). The same can be seen in the case of the asymptomatic vital tooth with a deep carious lesion on 20 years old female patient (item number 12); again the dentists who had followed training in cariology in the last 5 years selected more frequently to perform partial caries removal (OR = 5.1; 95% C.I. = 1.0 – 25.8; *p* = 0.046). This finding agrees with the results of Koopaeei *et al.* (5), who found that dentists stating more frequently reading articles and attending continuing education courses about minimally invasive treatments and cariology were more likely to be more conservative in their approach to management of DCLs.

In the two firsts proposed cases, the carious lesions not reached the inner third of the dentin, with no risk of pulp exposure. According to the ICCC group, selective removal to firm dentin is the treatment of choice (7). Although in the three cases there were no symptoms or signs of irreversible pulpitis associated with the DCL, being the diagnosis reversible pulpitis, in the third case periapical radiograph showed that lesion reached the inner quarter of the dentin. Growing evidence indicates that partial excavation, with selective removal to soft dentin, is the most appropriate treatment option for this type of DCLs (6,7), in which operative treatment implies high risk of pulp exposure. Peripheral enamel and dentin must be removed until feel hard dentin, ensuring a good seal and placement of the restoration (5,6,7,16), and soft carious dentin is left over the pulp, reducing the risk of pulp exposure (6,7). The ICCC group stated that “carious tissue is removed purely to create conditions for long-lasting restorations… bacterially contaminated or demineralized tissues close to the pulp do not need to be removed” (7). The bacteria still present in the soft dentin are entombed and without access to nutrients, modifying the bacterial flora and stopping the advance of the caries, with rehardening the soft dentin (17). It has been demonstrated that leaving infected dentin does not imply that caries progresses or that pulpitis or pulpal necrosis occurs (18). Radiographic follow-up during 10 years of carious lesions treated with selective removal to soft dentin showed deposition of tertiary dentin and increased radiopacity of the carious dentin left in the cavity floor (19). Definitely, for DCLs the periphery should be excavated removing all carious tissues until firm dentin, whilst for pulpal areas, soft dentin might be left to avoid exposure (3).

Regarding the knowledge and factors underlying their excavation strategy, when asked if cariogenic microorganisms must be removed or caries would progress, 50.4% strongly agreed, in accordance with the 47.2% of total disagreement to question if a certain amount of microorganisms could be left during caries removal. Dentists teaching at the University significantly agreed with both statement, with significant OR values for the first (OR = 4.6; 95% C.I. = 1.3 – 15.8; *p* = 0.017) and the second (OR = 4.5; 95% C.I. = 1.2 – 16.8; *p* = 0.0247) questions, respectively. This indicates that university professors are aware of new concepts about caries, suggesting that current dental students are receiving the correct information about cariology in their dental studies. If this is the case, it is to be expected that future Spanish dentists will incorporate into their practice the new minimally invasive concepts in the treatment of caries lesions that, currently, seem not to be followed by dental practitioners. The idea that carious tissue must be excavated to eliminate bacteria, which today has no scientific support, is correlated with the radical and non-conservative attitude adopted in the face of carious lesions. Similar finding was found by Schwendicke *et al.* (10). In the study by Weber *et al.* (12) in Brazil, 8.8% of 54 dentist respondents (out of 122 surveyed) would use partial caries removal for the management of DCLs. In a German study by Schwendicke *et al.* (10), dentists who believed residual caries was harmful tended to reject incomplete excavation of carious tissue, and those who felt it was acceptable favored indirect pulp-capping procedures. These authors found that 50% of dentists considered only complete caries excavation, even if pulp exposure was likely. In a survey concerning the management of DCLs. Oen *et al.* (21) showed that only 20% of GDs chose conservative treatment for DCLs.

Few studies have investigated the reasons and factors influencing the treatments decisions of dental practitioners treating DCLs. In the present study “good clinical result” (82%) was the main reason in the treatment decision-making process. This finding agrees with that reported by Stangvaltaite *et al.* (11), who found that “good results” (85%) were the most frequently reason for the selection of a DCL treatment. Regarding patient-related factors, patient’s oral health (84%) and patient´s age (70%) were the main factors in the treatment decision-making process. Again, these results are in accordance with the findings of Stangvaltaite *et al.* (11), who also found that “patient’s oral health” and “patient´s age” were main patient-related factors among those who chose vital pulp therapy of carious exposures. These responses are justified by studies showing lower success rate of vital pulp therapy in adult patients (22). However, the use of bioactive endodontic cements, such as MTA, in vital pulp therapy has shown favorable clinical and radiographic results regardless of the age of the patients (23). It is to be expected that the new evidence showing high success rates of vital pulp therapy in adults make age no longer a key factor in the choice of treatment in DCLs. In fact, a recent study investigating the preferred management methods of Finish dentists for DCLs in adult patients (24), has found that less invasive treatments are being selected into clinical practice by the majority of dentists in Finland. Similar results have been reported in a survey carried out to a sample of Spanish dentists (25).

## Conclusions

There is no uniform treatment method of teeth with DCLs and reversible pulpitis among the dentists included in the sample study. Total caries excavation is yet the chosen treatment in case of DCL with reversible pulpitis. Few dentists chose partial caries removal and a high percentage of them think that cariogenic microorganisms must be removed or caries would progress. The joint assessment of the answers given by respondents allows to conclude that the new knowledge and concepts about caries lesions and the more conservative approach to DCLs have not yet been incorporated by some Spanish dentists into their usual clinical practice. Although dental professionals have plenty access to scientific knowledge, this knowledge is not being translated into daily clinical practice.
